# Staufen Negatively Modulates MicroRNA Activity in *Caenorhabditis elegans*

**DOI:** 10.1534/g3.116.027300

**Published:** 2016-02-23

**Authors:** Zhiji Ren, Isana Veksler-Lublinsky, David Morrissey, Victor Ambros

**Affiliations:** * Program in Molecular Medicine, RNA Therapeutics Institute, University of Massachusetts Medical School, Worcester, Massachusetts, 01605; † Intellia Therapeutics Inc., Cambridge, Massachusetts 02139

**Keywords:** microRNA, *stau-1*, RNAi, RNA-binding protein, 3′UTR

## Abstract

The double-stranded RNA-binding protein Staufen has been implicated in various posttranscriptional gene regulatory processes. Here, we demonstrate that the *Caenorhabditis elegans* homolog of Staufen, STAU-1, functionally interacts with microRNAs. Loss-of-function mutations of *stau-1* significantly suppress phenotypes of *let-7* family microRNA mutants, a hypomorphic allele of *dicer*, and a *lsy-6* microRNA partial loss-of-function mutant. Furthermore, STAU-1 modulates the activity of *lin-14*, a target of *lin-4* and *let-7* family microRNAs, and this modulation is abolished when the 3′ untranslated region of *lin-14* is removed. Deep sequencing of small RNA cDNA libraries reveals no dramatic change in the levels of microRNAs or other small RNA populations between wild-type and *stau-1* mutants, with the exception of certain endogenous siRNAs in the WAGO pathway. The modulation of microRNA activity by STAU-1 does not seem to be associated with the previously reported enhanced exogenous RNAi (Eri) phenotype of *stau-1* mutants, since *eri-1* exhibits the opposite effect on microRNA activity. Altogether, our results suggest that STAU-1 negatively modulates microRNA activity downstream of microRNA biogenesis, possibly by competing with microRNAs for binding on the 3′ untranslated region of target mRNAs.

MicroRNAs (miRNAs) are a class of endogenous noncoding small RNAs that posttranscriptionally regulate gene expression, primarily through binding to the 3′ untranslated region (3′UTR) of target mRNAs and inhibiting translation and/or mRNA stability (He and Hannon 2004). miRNAs are usually transcribed into primary transcripts (pri-miRNAs) from independent genes by RNA polymerase II. Pri-miRNAs are processed into hairpin structured precursor miRNAs (pre-miRNAs) by the Drosha-DGCR8 complex in the nucleus, and then pre-miRNAs are exported into the cytoplasm and further processed by Dicer to generate the ∼21 nucleotide-long mature miRNAs (Krol *et al.* 2010). The seed sequence (nucleotides 2–7) of a mature miRNA dictates the specificity of a miRNA’s recognition of target mRNAs. Therefore, miRNAs with the same seed sequence are grouped into a family and are predicated to potentially share the same set of target mRNAs (Bartel 2009).

MiRNAs exert their repression on mRNAs through the assembly of the miRNA-induced silencing complex (miRISC) on the 3′UTR of target mRNAs. MiRISC is a ribonucleoprotein complex with a miRNA-specific Argonaute (AGO) protein loaded with a mature miRNA, and an AGO binding partner GW182 protein (Fabian and Sonenberg 2012). Besides AGO and GW182, other RNA-binding proteins have been shown to affect miRNA activity through biogenesis, such as the case of LIN-28 (Viswanathan and Daley 2010), miRISC activity, as for NHL-2 (Hammell *et al.* 2009), and target site accessibility, as shown for Pumilio, HuR, and Dnd1 (Nolde *et al.* 2007; Kedde *et al.* 2010, 2007; Bhattacharyya *et al.* 2006; Tominaga *et al.* 2011; Kundu *et al.* 2012; Young *et al.* 2012).

Staufen is a conserved double-stranded RNA-binding protein that contains five double-stranded RNA-binding domains, and was first identified in *Drosophila* to regulate mRNA localization and translation (*oskar* in oocytes, *bicoid* in embryos, and *prospero* in neuroblasts) (St Johnston *et al.* 1991; Li *et al.* 1997; Broadus *et al.* 1998). For example, Staufen binding to the 3′UTR of *bicoid* and *prospero* mRNAs is required for their localization (Ferrandon *et al.* 1994, 1997; Shen *et al.* 1997). In mammalian neurons, Staufen homologs (Staufen1 and Staufen2) are also known to regulate mRNA transport and the activation of localized mRNA translation (Köhrmann *et al.* 1999; Kiebler *et al.* 1999). Two groups have shown that Staufen1 can bind to long-range duplexes in the 3′UTR of mRNAs (Ricci *et al.* 2014; Sugimoto *et al.* 2015). Besides regulating mRNA localization and translation, mammalian Staufen can also mediate mRNA decay through interaction with the nonsense medicated decay regulator Upf1 (Park and Maquat 2013). In *Caenorhabditis elegans*, there is only one Staufen homolog, *stau-1*, and it has been shown to have high binding affinity for double-stranded RNA *in vitro*. STAU-1 is expressed at all developmental stages in *C. elegans*, and partial loss-of-function mutants [*stau-1(tm2266)* and *stau-1(q798)*] exhibit phenotypes that include enhanced transgene silencing, enhanced exogenous RNAi, and mild germline defects (LeGendre *et al.* 2013).

Previous studies have demonstrated that miRISC components and miRNAs are present in Staufen-containing RNA granules (Barbee *et al.* 2006; Peredo *et al.* 2014), which indicates that Staufen might affect the miRNA pathway, perhaps by influencing miRNA biogenesis and/or function. Here, we report genetic evidence that *C. elegans* STAU-1 exerts activity in opposition to certain miRNAs; we show that loss of function for *stau-1* genetically suppresses the phenotypes of mutants in several distinct miRNA genes and of a *Dicer* (*dcr-1*) mutant. Further, our small RNA sequencing data show that STAU-1 does not have any significant effect on the levels of mature miRNAs, indicating that Staufen can inhibit the activity of miRNAs downstream of miRNA biogenesis. Finally, we report data suggesting that Staufen-mediated opposition of miRNA activity acts via the 3′UTR of miRNA target mRNAs.

## Materials and Methods

### Nematode methods and phenotypic analysis

*C. elegans* were cultured on nematode growth media (NGM) (Brenner 1974) and fed with *Escherichia coli* HB101. All the *C. elegans* strains used in this study are listed in Supplemental Material, Table S11. Synchronized populations of developmentally staged worms were obtained by standard methods (Stiernagle 2006).

For heterochronic phenotype analysis, gravid adult animals raised at 20° were placed on NGM plates seeded with *E. coli* HB101 at 20°, unless otherwise noted, and their progeny were scored at the young adult stage for adult lateral alae formation and seam cell number. Nomarski DIC microscopy was used to score alae formation, and fluorescence microscopy with the *maIs105* [*col-19*::*gfp*] or *wIs51* [*scm*::*gfp*] transgenes to mark lateral hypodermal cell nuclei was used to score seam cell number.

The ASE neuron phenotype was scored by the expression of *otIs114* [*lim-6*::*gfp*] (ASEL marker) in the larvae of each genotype using a Zeiss SteREO Discovery.V12 microscope (Hammell *et al.* 2009).

### Targeted genome editing by CRISPR/Cas9

In order to generate *stau-1* null mutants, we adapted previously described co-CRISPR strategies (Kim *et al.* 2014; Arribere *et al.* 2014) with our modifications. Wild type animals (N2) were injected with a mixture containing 40 ng/μl *eft-3*::*cas9* vector, 35 ng/μl *unc-22* sgRNA vector, 35 ng/μl *dpy-10* sgRNA vector, 35 ng/μl *stau-1* sgRNA-1 vector, 35 ng/μl *stau-1* sgRNA-2 vector, and 15 ng/μl *sur-5*::*gfp* vector. The sequences for *stau-1* sgRNAs were: 5′-GGATGGAGTGATGATAGTAC-3′ (sgRNA-1) and 5′-TACGGATCTGGCAGATACTT-3′ (sgRNA-2). F1 worms exhibiting any of the dumpy and/or twitching phenotypes, and/or *sur-5*::*gfp* expression, were picked individually to plates and allowed them to produce F2 progeny. These F1 animals were lysed in 10 μl single-worm lysis buffer (50 mM KCl, 10 mM Tris-HCl pH 8.2, 2.5 mM MgCl_2_, 0.45% NP-40, 0.45% Tween-20, 0.01% Gelatin, and 60 ng/μl proteinase K) at 60° for 1 hr. PCR reactions were performed with primers (5′-TCCTTCAATCGATGTGGCCAA-3′ and 5′-TGGCTCACATTTTGTTAAACGACA-3′) and the sequence of PCR products was determined using Sanger sequencing. Both of the two mutations recovered were from CRISPR/Cas9 editing events by sgRNA-1.

### Western blot analysis

For STAU-1 western blots, samples were prepared from populations of mixed stage embryos and synchronized L4 stage larvae grown on *E. coli* HB101 at 20°. Animals were washed off plates with M9 buffer and flash frozen in liquid nitrogen. Lysates were prepared by resuspending samples in lysis buffer [25 mM HEPES pH 7.5, 100 mM NaCl, 0.25 mM EDTA, 0.1% NP-40, 2 mM DTT, PhosSTOP (Roche), and Protease inhibitor (Roche)] and homogenized with a Branson SLPe sonicator. Lysates were centrifuged at 164,000 rpm for 15 min at 4° and the supernatants were collected. BioRad Protein Assay Dye Reagent Concentrate (Cat# 500-0006) was used to measure the protein concentration. 80 μg of protein were used for the immunoblot analysis. STAU-1 was recognized by an antiserum generated in the laboratory of Dr. Marvin Wickens (LeGendre *et al.* 2013) at 1:1000 dilution, gel loading was calibrated by reprobing blots with anti-α-tubulin antibody (Sigma-Aldrich Cat# T6074) at 1:20,000 dilution.

### RNA extraction and small RNA cDNA cloning

Wild type (N2) and *stau-1(tm2266)* young adults were collected and flash frozen in liquid nitrogen. Three biological replicates were analyzed for each strain. Total RNA was extracted using Trizol reagent (Invitrogen). 20 μg of total RNA for each sample was used to isolate small RNA populations. RNA samples were run on a 15% PAGE/urea gel and small RNA populations were isolated from the gel with sizes ranging from 18–26 nucleotides (nt). A previously published small RNA cloning protocol (Sterling *et al.* 2015) was used to generate cDNA libraries with the following modifications: 1) 3′ ligation reactions were performed at 4° overnight; 2) 100 units of Superscript III Reverse Transcriptase were used for first-strand cDNA synthesis for each sample and the Reverse Transcriptase reaction was performed at 42° for 90 min. The Sterling *et al.* (2015) protocol involves only one RNA ligation to the 3′ end of the RNA, and hence recovers RNA species regardless of 5′ end structure.

### Computational analysis of small RNA libraries

cDNA libraries were sequenced on the Ion Torrent (Proton) instrument according to manufacturer’s protocols. Sequencing files in FastQ formats were processed using the Cutadapt method (version 1.2.1) (Martin 2011) to remove the adapter sequences with the following options -e 0.25 -g 5′-ATTGATGGTGCCTACAG-3′ -a 5′-GATCGTTCGGACTGTAGATC-3′.

Sequence files were split into libraries according to barcode sequences, and reads shorter than 16 nt were removed. For each library, reads with identical sequences were combined and the combined count was saved in Fasta files. Reads were then aligned to the *C. elegans* genome (WormBase release WS215) using bowtie (Langmead *et al.* 2009) with arguments, -v 3 -f -B 1 -a–best –strata. Alignments were then filtered based on the length of the reads and the number of mismatches as follows: for sequence lengths 16–17, 18–19, 20–24, or >24: zero, one, two, or three mismatches were allowed, respectively.

Annotations of coding genes, transposons, tRNAs, rRNAs, piRNAs, and miRNAs were obtained from WormBase (release WS215) and miRBase (Griffiths-Jones *et al.* 2008) (release 20). An in-house developed code was used to analyze the mapping results. To assign read counts to the miRNA sequences, we considered all reads that mapped to the miRNA genomic loci starting within –5 to +5 nt of the annotated 5′ end of mature miRNAs. For all the other small RNA species and genomic features (*e.g.*, coding genes and transposons), we counted all reads that mapped within the annotated region in sense and antisense orientations separately.

For endo-siRNA analysis, we considered all reads that mapped antisense to the 5′UTR, coding exons, and 3′UTR regions of each annotated gene. Annotations of target genes in the CSR-1, WAGO, ALG-3/4, and ERGO-1 pathways were downloaded from (Lee *et al.* 2012).

Differential expression analysis was performed using the edgeR package in R (Robinson *et al.* 2010).

### Data availability

Sequence data files are available in the GEO database under the accession number GSE79217. Additional methods are described in File S1.

## Results

### STAU-1 functionally modulates the activity of several miRNAs

In order to identify modulators of miRNA activity, we established a panel of worm strains containing mutations designed to produce sensitized genetic backgrounds with compromised activity of specific miRNA families or miRNA biogenesis factors. There are three categories of mutations in these sensitized genetic backgrounds: 1) null mutations of a subset of genes encoding a miRNA family; 2) hypomorphic (nonnull partial loss-of-function) mutations of a particular miRNA; 3) hypomorphic mutations of a miRNA biogenesis factor or miRISC component. One essential feature of the sensitized genetic backgrounds is that these mutants all have partially penetrant phenotypes. This feature allows the identification of either positive or negative modulators of miRNA activity by testing for enhancement or suppression, respectively, of these sensitized phenotypes after genetic or RNAi knockdown of candidate gene activity.

**Figure 1:**
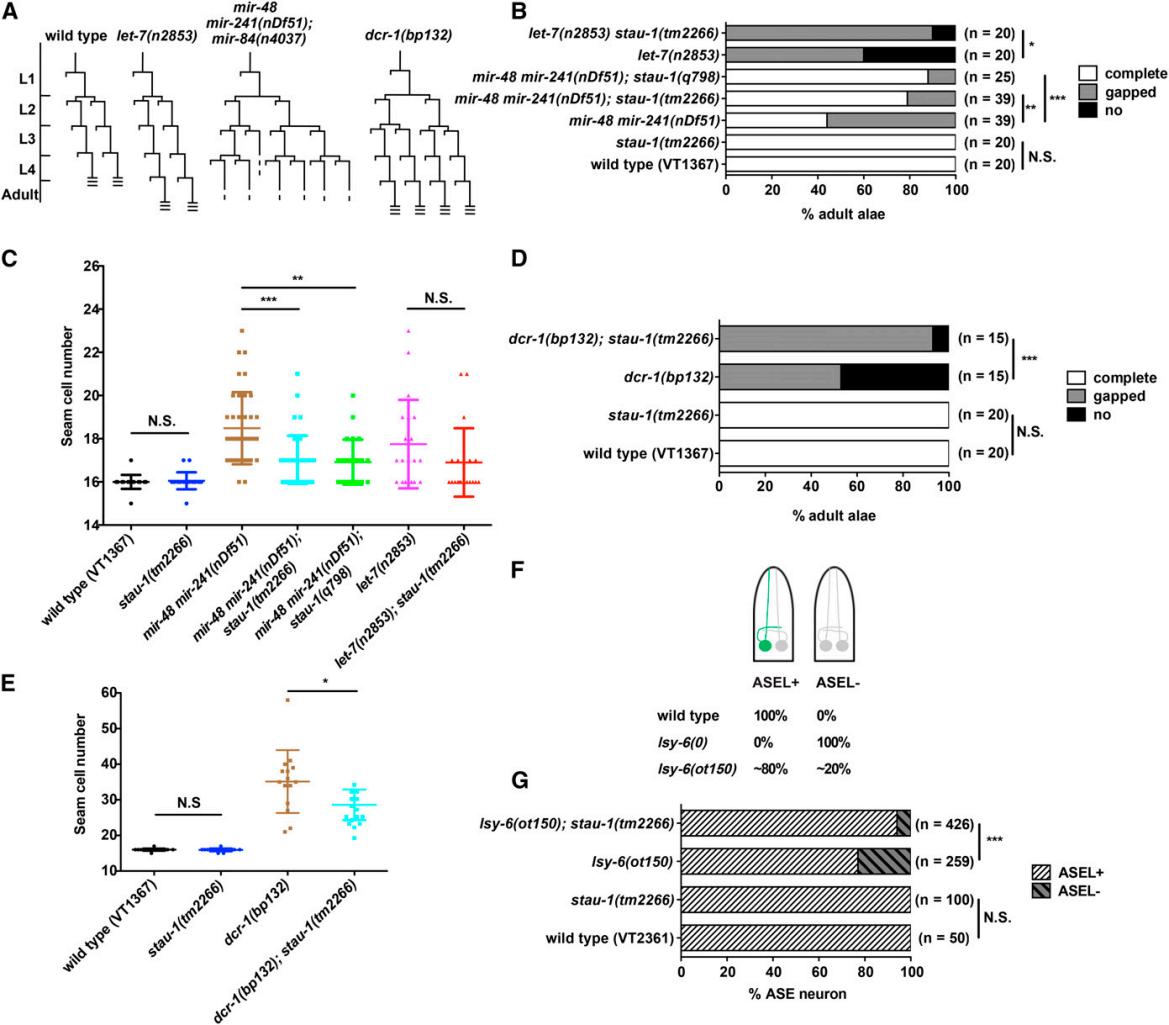
Loss of function for *stau-1* suppresses the phenotypes associated with mutations of genes encoding miRNAs, or DCR-1, a miRNA biogenesis factor. (A) Diagrams of seam cell V lineage in wild type (N2), *let-7(n2853)*, *mir-48 mir-241(nDf51)*; *mir-84(n4037)*, and *dcr-1(bp132)* animals. L1 to L4 are the four larval stages in *C. elegans* postembryonic development. The three horizontal lines indicate adult alae formation. (B) Adult alae and (C) seam cell phenotypes of *stau-1* mutants in wild type and *let-7* family miRNA mutant backgrounds. (D) Adult alae and (E) seam cell phenotypes of *stau-1(tm2266)* in combination with *dcr-1(bp132)*. The experiment with *dcr-1(bp132)* was carried out at 15°. (F) A diagram of *C. elegans* larvae illustrating the ASE (amphid sensilla) neuron phenotype of miRNA *lsy-6* mutants. The green fluorescent protein (GFP) expression in ASEL (amphid sensilla, left) neuron is driven by *lim-6* promoter. *lsy-6(0)* indicates the null allele of *lsy-6*; *lsy-6(ot150)* is a partial loss-of-function mutation. (G) ASE neuron phenotype of *stau-1(tm2266)* and double mutant of *stau-1(tm2266)*; *lsy-6(ot150)*. miRNA, microRNA; N.S., not significant (chi-square test for adult alae phenotype and ASE neuron phenotype, two-tailed *t*-test for seam cell phenotype); * *P* < 0.05; ** *P* < 0.01; *** *P* < 0.001.

The first sensitized genetic backgrounds we investigated were the *let-7* family miRNA mutants. *C. elegans let-7* family miRNAs (including *let-7*, *mir-48*, *mir-84*, and *mir-241*) function semiredundantly in controlling the developmental timing of certain stage-specific hypodermal seam cell fates. Loss of *let-7* family miRNAs results in reiterations of early larval seam cell division patterns at later stages, and seam cells in these mutants also fail to properly differentiate adult specific cuticular structures (called adult alae) at the young adult stage (Figure 1A). Three of the *let-7* family miRNAs (*mir-48*, *mir-84*, and *mir-241*) are expressed starting at the L2 stage and function to regulate the L2 stage proliferative seam cell division, while the *let-7* miRNA is strongly upregulated from the L3 stage to control the larval-to-adult transition of seam cells (Reinhart *et al.* 2000; Abbott *et al.* 2005). These heterochronic *let-7* family miRNA mutant phenotypes are easily quantified by using microscopy to measure the formation of adult alae and to score the number of seam cells in young adults.

To test if STAU-1 modulates *let-7* family miRNA activity, we used two mutant strains [*mir-48mir-241(nDf51)* and *let-7(n2853)*], which both have partially penetrant heterochronic phenotypes with gaps in adult alae and increased number of seam cells at the young adult stage. *mir-48mir-241(nDf51)* mutant has two *let-7* family miRNAs removed while *let-7(n2853)* is a strong loss-of-function mutation at the seed region of *let-7* mature miRNA (Reinhart *et al.* 2000). Although the *stau-1* loss-of-function mutant does not exhibit any developmental timing defects in an otherwise wild-type genetic background, we observed that both *stau-1(tm2266)* and *stau-1(q798)* significantly suppresses the heterochronic phenotypes of the *mir-48mir-241(nDf51)* mutant (Figure 1, B and C). Since *stau-1(tm2266)* and *stau-1(q798)* have similar effects on the phenotypes of *mir-48mir-241(nDf51)* animals, we focused only on *stau-1(tm2266)* for further analysis. Besides suppressing the heterochronic phenotypes of *mir-48mir-241(nDf51)* animals, *stau-1(tm2266)* also exerts significant suppression of the heterochronic adult alae phenotype of *let-7(n2853)* animals (Figure 1B). We interpret this suppression of heterochronic phenotypes of *let-7* family miRNA mutants by *stau-1* loss of function to suggest that loss of *stau-1* function causes an increase in the activity of the remaining *let-7* family miRNAs. These results indicate that STAU-1 acts as a negative modulator of *let-7* family miRNA biogenesis or activity.

The second sensitized genetic background we tested is a *dicer* (*dcr-1*) hypomorphic allele, *bp132*. This mutation causes a single amino acid change in the RNase III domain of DCR-1 and has been previously shown to cause developmental timing defects, as indicated by an increased number of seam cells and failure to form complete adult alae at the young adult stage (Figure 1A). The phenotypes of this partial loss-of-function mutant *dcr-1(bp132)* are stronger at 15° and can be suppressed by a point mutation that alters the sequence of the *lin-4* miRNA precursor (Lee *et al.* 1993; Wightman *et al.* 1993; Ren and Zhang 2010), indicating that the *dcr-1(bp132)* phenotypes reflect partially compromised *lin-4* biogenesis. We found that *stau-1(tm2266)* suppresses both adult alae and seam cell phenotypes of this *dcr-1(bp132)* mutant at 15° (Figure 1, D and E), suggesting that STAU-1 negatively modulates *lin-4* biogenesis or activity.

The third sensitized genetic background that we employed is a *lsy-6* miRNA hypomorphic mutant. *lsy-6* is known to regulate the asymmetric cell fate decision in ASE neurons (Johnston and Hobert 2003). The null allele of *lsy-6* causes a highly penetrant cell fate transformation phenotype, where the ASEL neuron adopts the cell fate of the ASER neuron, which is detected by loss of expression of the ASEL marker *lim-6*. The *lsy-6(ot150)* allele is a nonnull (hypomorphic) point mutation 111 nt upstream of the *lsy-6* hairpin, which disrupts a *cis*-regulatory element required for *lsy-6* expression. The *lsy-6(ot150)* animals exhibit a weak phenotype with ∼20% penetrance (Sarin *et al.* 2007) (Figure 1F). *stau-1(tm2266)* animals do not exhibit any ASE neuron cell fate defects since all the animals have *lim-6* expression only in the ASEL neurons, yet the phenotype of *lsy-6(ot150)* animals is significantly suppressed by *stau-1(tm2266)* (Figure 1G). These results indicate that loss of function of *stau-1* can potentiate the activity of *lsy-6*, suggesting that the role of STAU-1 as a negative modulator of miRNA activity is not restricted to miRNAs of the heterochronic pathway.

### stau-1 null mutants have an effect on miRNA activity similar to that of partial loss-of-function mutants

**Figure 2:**
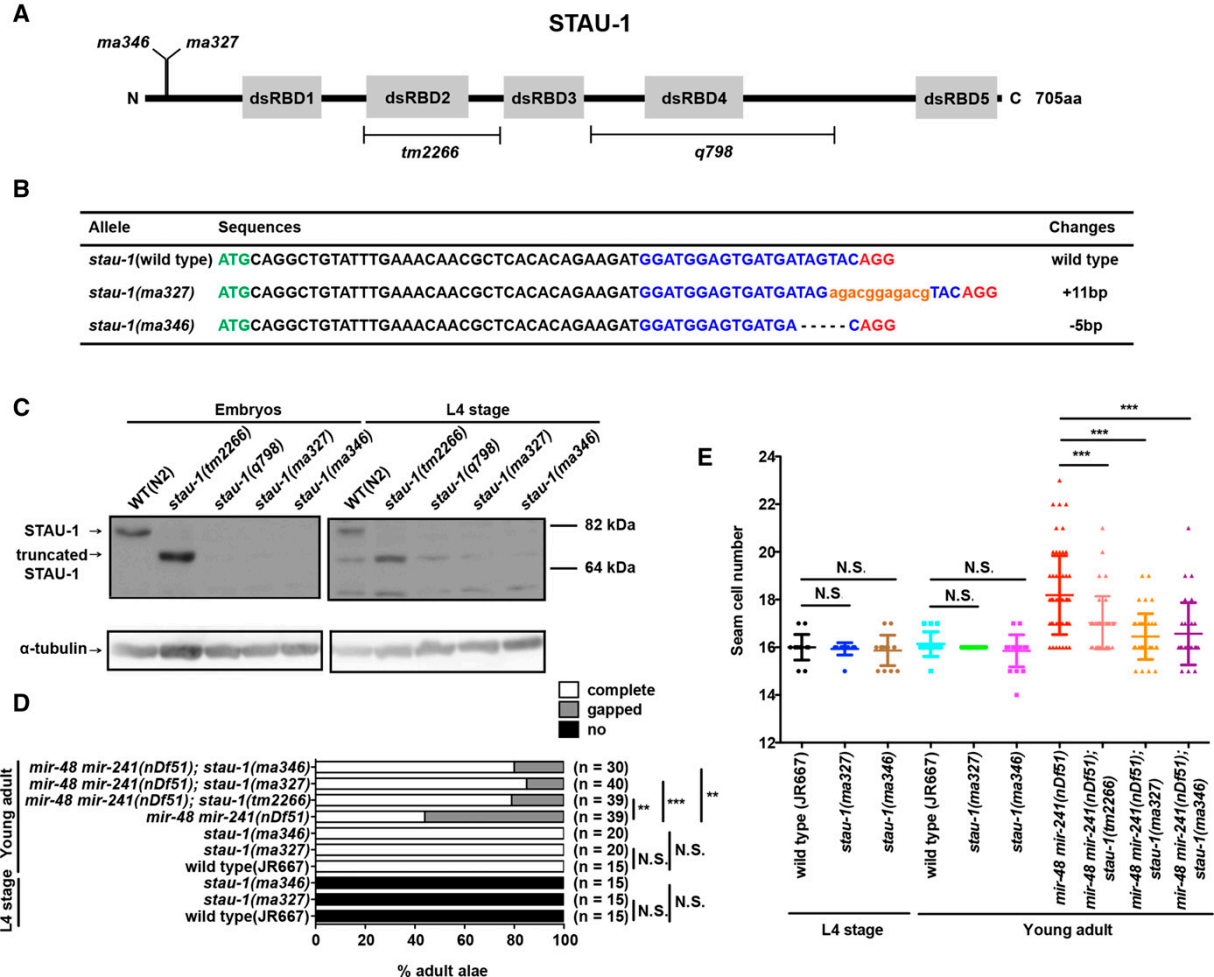
Characterization of *stau-1* null alleles. (A) A diagram of protein domains and mutations of STAU-1 used in this study. The five double-stranded RNA-binding domains (dsRBDs) are illustrated as gray boxes. The deletions in *stau-1(tm2266)* and *stau-1(q798)* (LeGendre *et al.* 2013) are shown by brackets. The positions of *ma327* and *ma346* mutations are indicated. (B) Nucleotide changes of *stau-1(ma327)* and *stau-1(ma346)* mutants. All the sequences shown here are in the beginning of *stau-1*’s first exon. The ATG start codon is in green. The sgRNA sequence is highlighted in blue and the PAM sequence is in red. The inserted sequence in *ma327* is in orange and lower case. The premature termination codons in *ma327* and *ma346* are 89 and 65 amino acids downstream from the N-terminus respectively. (C) Western blots of STAU-1 and α-tubulin in wild type and *stau-1* mutants. Both mix stage embryos and L4 stage animals were used for this experiment. There are nonspecific bands in the L4 stage blot. (D) Adult alae and (E) seam cell phenotypes of the *stau-1* null mutants at the L4 and the young adult stage in wild type and *mir-48 mir-241(nDf51)* backgrounds. ** *P* < 0.01; *** *P* < 0.001; N.S., not significant (chi-square test for adult alae phenotype and two-tailed *t*-test for seam cell phenotype); sgRNA, single guide RNA.

The *stau-1* mutants available so far are partial loss-of-function mutants that remove either the second (*tm2266*) or the fourth (*q798*) double-stranded RNA-binding domain (Figure 2A). Therefore, to test the effect of *stau-1* null mutants on miRNA activity and to determine if *stau-1* may have additional functions, we carried out CRISPR/Cas9 experiments to generate null mutants of *stau-1*. The guide RNA was designed to target the first exon of *stau-1* and we screened for frameshift mutations that lead to premature stop codons. Two independent mutations were isolated: *ma327*, an 11 bp insertion, and *ma346*, a 5 bp deletion, both of which generate early premature stop codons (Figure 2, A and B).

To confirm that *ma327* and *ma346* mutations are null alleles, we tested for expression of STAU-1 protein in these mutant animals. We performed western blot analysis of wild type and *stau-1* mutant embryos and L4 stage larvae using an antiserum generated against the fourth double-stranded RNA-binding domain of STAU-1 (LeGendre *et al.* 2013) (Figure 2C). *stau-1(q798)* animals lacking the fourth double-stranded RNA-binding domain were used as negative controls. As expected, the *stau-1(tm2266)* mutation resulted in a truncated STAU-1 protein recognizable by the antiserum. In the embryos, we only observed full-length and truncated STAU-1 in wild type animals and *stau-1(tm2266)* mutants, respectively, whereas neither *stau-1(ma327)* nor *stau-1(ma346)* embryos contained any detectable STAU-1 protein. In the L4 stage samples, there were nonspecific bands close to the size of the truncated STAU-1, but the *stau-1(ma327)* and *stau-1(ma346)* mutants had the same band pattern as the negative control. Therefore, the *stau-1* alleles *ma327* and *ma346* generated in this study appear to be null alleles.

Next, we sought to characterize the phenotypes of these *stau-1* null alleles. Homozygous mutants of either *ma327* or *ma346* are viable. However, similar to *tm2266* and *q798*, these animals exhibit a 4 hr delay in larval development at 20° and they are smaller in size compared to wild type animals at the young adult stage (Figure S1). Furthermore, to test if the *stau-1* null mutants also suppress phenotypes of miRNA mutants, we crossed the null alleles into the *let-7* family mutant *mir-48mir-241(nDf51)*. As expected, both of these null alleles significantly suppressed the adult alae and seam cell phenotypes of *mir-48mir-241(nDf51)* animals (Figure 2, D and E). Interestingly, neither *stau-1(ma327)* nor *stau-1(ma346)* animals exhibited any heterochronic defects in an otherwise wild-type genetic background (Figure 2, D and E), indicating that the *let-7* family hyperactivity that would result from the loss of STAU-1 was below the threshold required to elicit a precocious developmental timing phenotype.

Since the effects of these *stau-1* null alleles on *let-7* family miRNA activity are similar to the effect of *stau-1(tm2266)*, subsequent studies were conducted using *stau-1(tm2266)*.

### STAU-1 does not dramatically affect mature miRNA levels

**Figure 3:**
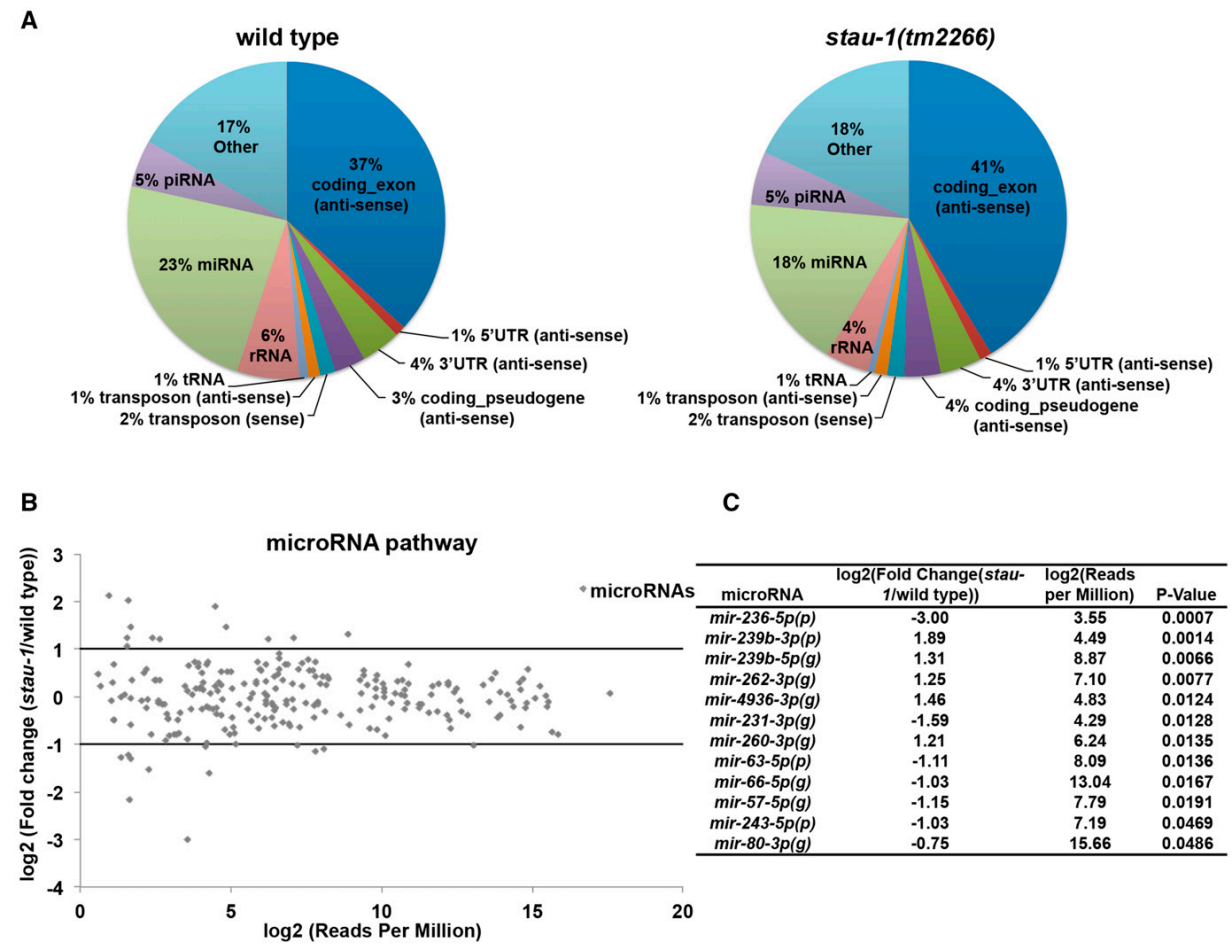
STAU-1 does not significantly affect mature miRNA levels. (A) Read distribution of small RNAs mapped to *C. elegans* genome in wild type and *stau-1(tm2266)* animals. All the antisense reads are endo-siRNA reads since *C. elegans* endo-siRNAs are mapped antisense to various regions of different gene transcripts (Gu *et al.* 2009). Most of reads in “other” were mapped to the sense strand of protein coding genes. (B) Differential gene expression analysis of miRNAs between wild type and *stau-1(tm2266)* animals. (C) List of miRNAs whose levels are significantly affected in *stau-1(tm2266)* animals. (g), miRNA guide strand; (p), miRNA passenger strand.

To investigate the mechanism of how *stau-1* modulates miRNA activity, we first performed small RNA high-throughput sequencing analysis of wild type and *stau-1(tm2266)* animals at the young adult stage. Three replicate samples were sequenced for each genotype and all the replicates yielded more than 5,000,000 reads, with ∼80% of the reads mapping to the *C. elegans* genome. Since the small RNA cloning technique we used is not dependent on the structure of the 5′ nucleotide of the RNA (Sterling *et al.* 2015), we were able to examine STAU-1’s effect on diverse small RNA populations, including miRNAs, piRNAs, and endogenous siRNAs. Based on the reads distribution data of small RNA populations, we did not observe any dramatic difference between wild type and *stau-1(tm2266)* animals (Figure 3A).

Next, we focused our analysis on the levels of mature miRNAs. In our sequencing data, we identified a total of 239 miRNAs. However, only 12 miRNAs were significantly changed in *stau-1(tm2266)* compared to wild type animals (five upregulated and seven downregulated) (Figure 3, B and C, Table S1, and Table S2). None of the miRNAs that are differentially expressed in *stau-1(tm2266)* animals are known to be able to contribute to the phenotypes suppressed by *stau-1(tm2266)* (Figure 1). Additionally, since the precocious expression of *let-7* at the L2 stage, rather than the overexpression at the young adult stage, could affect the developmental timing phenotypes (Vadla *et al.* 2012), we examined the mature miRNA levels at the L2 stage. No significant change in mature miRNA levels was detected in either *stau-1(tm2266)* or *stau-1(q798)* animals compared to wild type animals (Figure S2). Consistent with these results, we did not observe any significant change in the protein levels of two miRNA biogenesis factors [DCR-1 and ALG-1 (a *C. elegans* AGO)] in several *stau-1* mutants (Figure S3). Therefore, we concluded that the modulation by STAU-1 of miRNA activity occurs downstream of miRNA biogenesis.

### STAU-1 does not dramatically affect other small RNA populations

**Figure 4:**
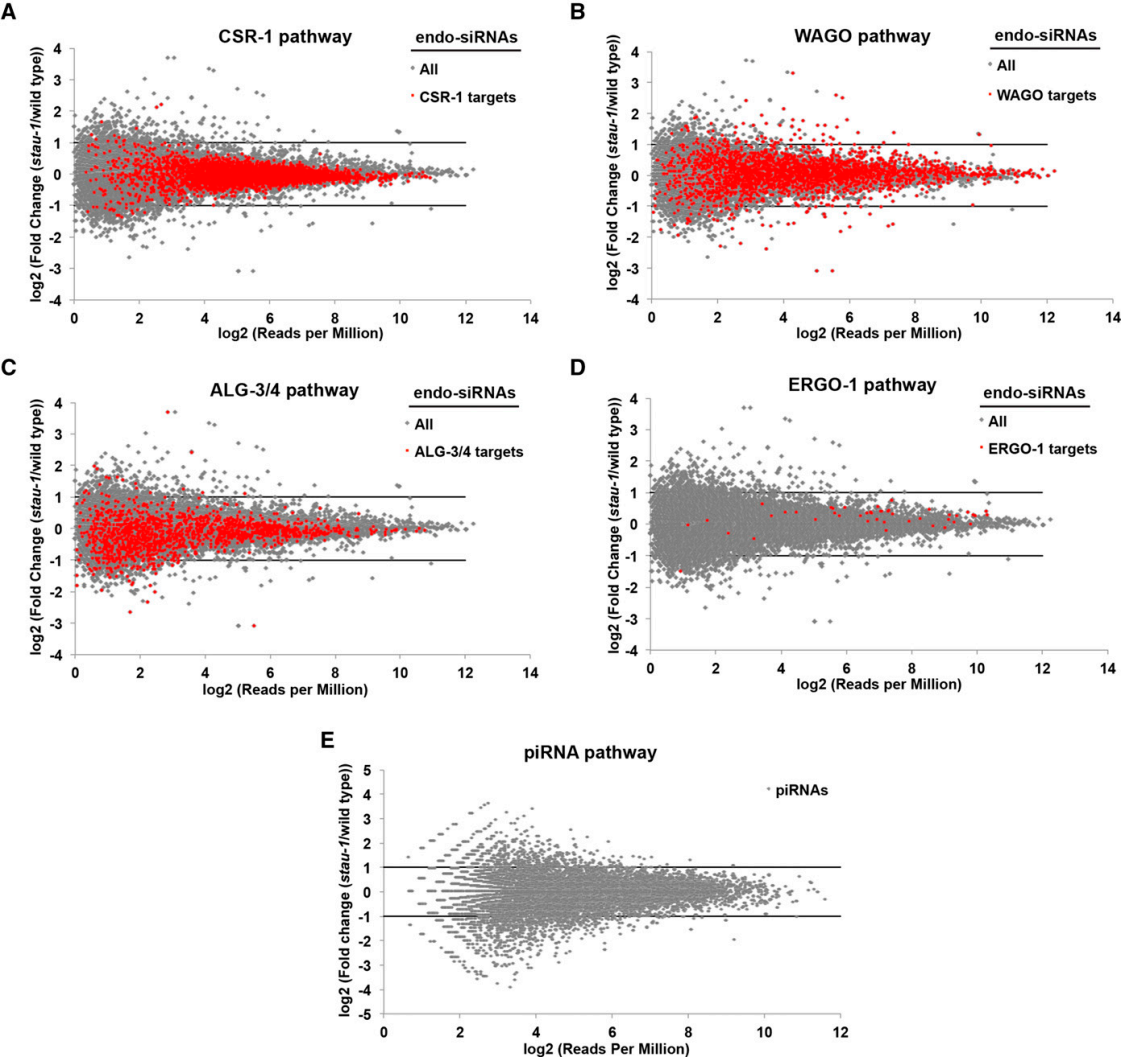
The effects of STAU-1 on small RNA pathways other than miRNAs. Comparisons between wild type and *stau-1(tm2266)* animals regarding expression of endo-siRNAs (A–D) associated with the CSR-1 (A), WAGO (B), ALG-3/4 (C), and ERGO-1 pathways (D), and piRNAs (PIWI-interacting RNAs) (E).

Since *stau-1* mutants exhibit enhanced RNAi and transgene silencing phenotypes (LeGendre *et al.* 2013), we examined whether there is any change in other small RNA populations between wild type and *stau-1(tm2266)* animals. Besides miRNAs, *C. elegans* also possesses several classes of endogenous siRNAs (endo-siRNAs) and piRNAs. Since different functional classes of endo-siRNAs are loaded into the distinct Argonaute proteins, CSR-1, WAGO, ALG-3/4, and ERGO-1 (Lee *et al.* 2012), we mapped our sequencing reads to these annotations and carried out differential gene expression analysis (Figure 4, A–D, Table S3, Table S4, Table S5, Table S6, Table S7, and Table S8). Of all these endo-siRNA categories, the only noteworthy changes we observed were for a subset of WAGO endo-siRNAs, which may function to maintain the silencing of “nonself” transcripts in *C. elegans* germline (Shirayama *et al.* 2012) (Figure 4B and Table S6). STAU-1 does not seem to simply promote biogenesis of endo-siRNAs in the WAGO pathway since cases of upregulation and downregulation of endo-siRNAs were evident in *stau-1(tm2266)* animals (Figure 4B). The final class of small RNAs that we analyzed was piRNAs, and there was no dramatic change in the piRNA levels between wild type and *stau-1(tm2266)* animals (Figure 4E, Table S9, and Table S10).

### The modulation of miRNA activity by *stau-1* mutations is likely independent of their enhanced RNAi phenotype

**Figure 5:**
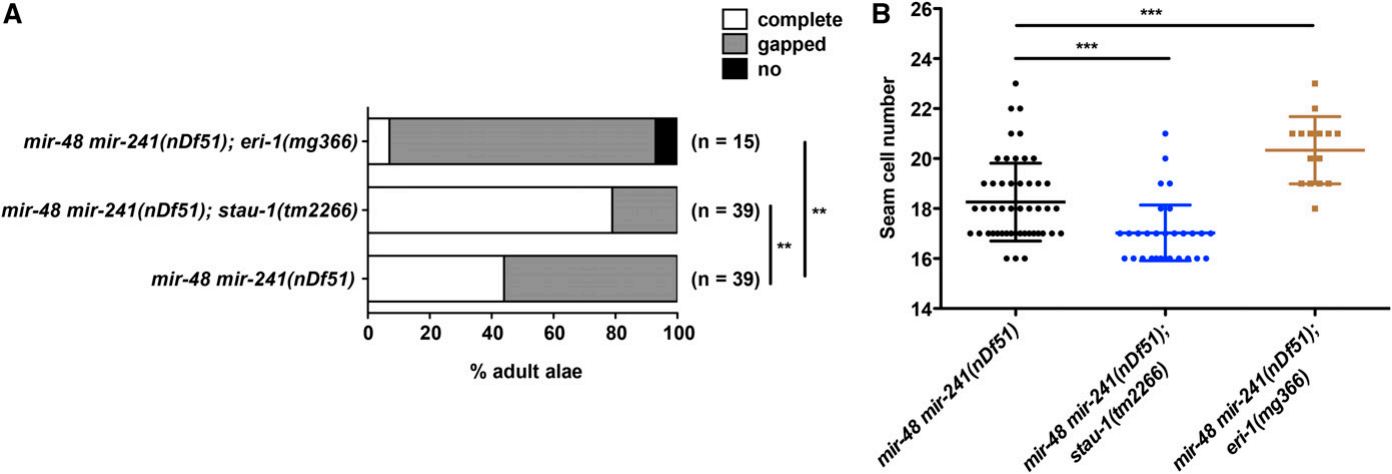
ERI-1 positively modulates *let-7* family miRNA activity. (A) Adult alae and (B) seam cell phenotype of *eri-1(mg366)* in combination with *mir-48 mir-241(nDf51)*. N.S., not significant ** *P* < 0.01; *** *P* < 0.001 (chi-square test for adult alae phenotype and two-tailed *t*-test for seam cell phenotype).

*C. elegans stau-1* mutants have been shown to exhibit an enhanced RNAi (Eri) phenotype, indicating that STAU-1 negatively modulates one or more RNAi pathways. This suggests that STAU-1’s negative modulation of miRNA activity that we have shown here could reflect a common underlying effect of STAU-1 on small RNA silencing more broadly. In that case, one might expect that other Eri mutants may also exhibit enhanced miRNA activity. Because *stau-1* mutants had been shown to interact genetically with *eri-1* (LeGendre *et al.* 2013), we tested whether *eri-1* loss-of-function could affect the heterochronic phenotypes of *mir-48mir-241(nDf51)* animals. Interestingly, loss of function of *eri-1* enhanced the adult alae and seam cell defects of *mir-48mir-241(nDf51)* animals (Figure 5), which is opposite to the suppression caused by *stau-1* mutation. Therefore, the modulation of miRNA activity by STAU-1 is unlikely to be simply the result of a general enhancement of RNA interference.

### STAU-1 may act through the 3′UTR of miRNA targets to modulate miRNA activity

Staufen has been shown to promote the translation of its target mRNAs (Micklem *et al.* 2000; Dugré-Brisson *et al.* 2005; Ricci *et al.* 2014), suggesting that the negative effect of STAU-1 on miRNA activity shown here could reflect its role in promoting the translation of miRNA target mRNAs, either by binding to 3′UTR sequences (perhaps at or near miRNA binding sites), or to other regions of the mRNAs (such as 5′UTR and/or coding sequences). To investigate whether STAU-1 could oppose miRNA activity relatively directly, via the 3′UTR sequences of the target mRNAs, we utilized mutants of the heterochronic miRNA *lin-4* and its primary target *lin-14* (Lee *et al.* 1993; Wightman *et al.* 1993).

**Figure 6:**
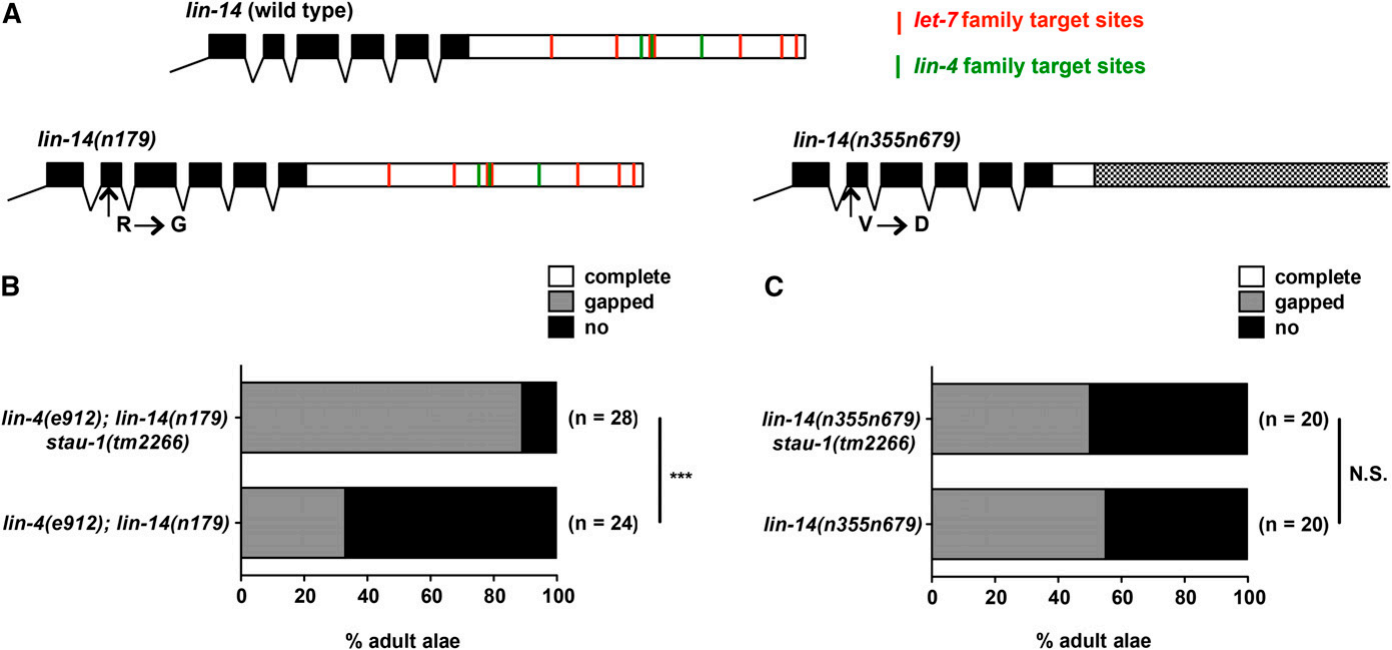
The 3′UTR of *lin-14* is required for modulation of *lin-14* gain-of-function phenotypes by *stau-1* mutation. (A) Diagrams of C-terminal end of *lin-14* gene in wild type, *lin-14(n719)*, and *lin-14(n355n679)*. The black boxes represent exons and the white boxes represent 3′UTR. The dotted box indicates the region of 3′UTR deleted in *lin-14(n355n679)*. The predicated *lin-4* family (green lines) and *let-7* family (red lines) target sites are indicated; the target site predication was obtained from TargetScan (Lewis *et al.* 2005; Jan *et al.* 2011). (B) Adult alae phenotype of *lin-4(e912)*; *lin-14(n179)* and *lin-4(e912)*; *lin-14(n179) stau-1(tm2266)* animals. (C) Adult alae phenotype of *lin-14(n355n679)* and *lin-14(n355n679) stau-1(tm2266)* animals. *** *P* < 0.001; N.S., not significant (chi-square test for adult alae phenotype and two-tailed *t*-test for seam cell phenotype).

The 3′UTR of *lin-14* possesses several *lin-4* and *let-7* family miRNA target sites (Figure 6A). The first strain we tested was *lin-4(e912)*; *lin-14(n179)*, which has a null mutation of *lin-4* and a point mutation in *lin-14*, and this double mutant exhibits a temperature sensitive heterochronic phenotype. At permissive temperature (15°), *lin-4(e912)*; *lin-14(n179)* animals exhibit a partially penetrant phenotype (∼30% animals have gaps in the adult alae) and can be considered a sensitized genetic background. *stau-1(tm2266)* significantly suppresses the adult alae phenotype of *lin-4(e912)*; *lin-14(n179)* animals (Figure 6B). This indicates that STAU-1 could modulate the activity of *lin-14*, possibly through *let-7* family miRNAs or *mir-237* (the other member of *lin-4* family miRNAs in *C. elegans*).

Next, we used another *lin-14* mutant strain *lin-14(n355n679)*. *n355* is a breakpoint mutation in the 3′UTR of *lin-14*, which results in the removal of most of the *lin-14* 3′UTR, including all the *lin-4* and *let-7* family miRNA binding sites (Shi *et al.* 2013). In combination with another point mutation (*n679*) that partially compromises LIN-14 function, the phenotype of *lin-14(n355n679)* is similar to *lin-4(e912)*; *lin-14(n179)* animals. However, we failed to observe any suppression by *stau-1(tm2266)* on the adult alae phenotype on *lin-14(n355n679)* animals (Figure 6C). These data suggest that STAU-1 may modulate miRNA activity through the 3′UTR of miRNA targets.

## Discussion

The double-stranded RNA-binding protein Staufen has been characterized as a master regulator of mRNA localization and translation in many metazoan species (Roegiers and Jan 2000). Staufen is known to positively regulate translation upon localization through interactions with structured regions of mRNAs (3′UTRs, coding sequences, and 5′UTRs) and various partner proteins and/or ribosomes (Ferrandon *et al.* 1994; Micklem *et al.* 2000; Dugré-Brisson *et al.* 2005; Ricci *et al.* 2014). Besides such positive roles in gene expression, Staufen can also negatively regulate gene expression by recruiting the nonsense-mediated decay factor Upf1 to the 3′UTR of mRNAs to trigger mRNA degradation (Park and Maquat 2013). Because of these alternative positive or negative roles in posttranscriptional regulation of mRNA activity, we predicted that Staufen could be expected to interact functionally with miRNA-mediated repression of mRNA targets, and could exert either promotion or inhibition of miRNA activity.

In this study, we found that loss-of-function Staufen (*stau-1*) mutations in *C. elegans* can suppress the phenotypes of miRNA partial loss of function, indicating that STAU-1 inhibits miRNA activity. This suggests that, at least with respect to the miRNAs whose functions we examined here, STAU-1 engages its translational enhancer function, rather than its mRNA decay activity. We show that *stau-1* loss-of-function mutation does not appreciably affect the levels of mature miRNAs; in particular, there was no detectable change, in *stau-1* mutants, of the levels of the *lin-4*, *let-7* family, and *lsy-6* miRNAs whose function we monitored phenotypically in our genetic interaction experiments. This strongly suggests that STAU-1 likely opposes the activity of these miRNAs independently of their biogenesis or turnover, and perhaps may act by binding to their target mRNAs. Consistent with a model where STAU-1 can modulate miRNA activity by binding to the 3′UTR of miRNA targets, our data show that the 3′UTR of a miRNA target, *lin-14*, is required for STAU-1-mediated modulation of *lin-14* heterochronic phenotypes.

**Figure 7:**
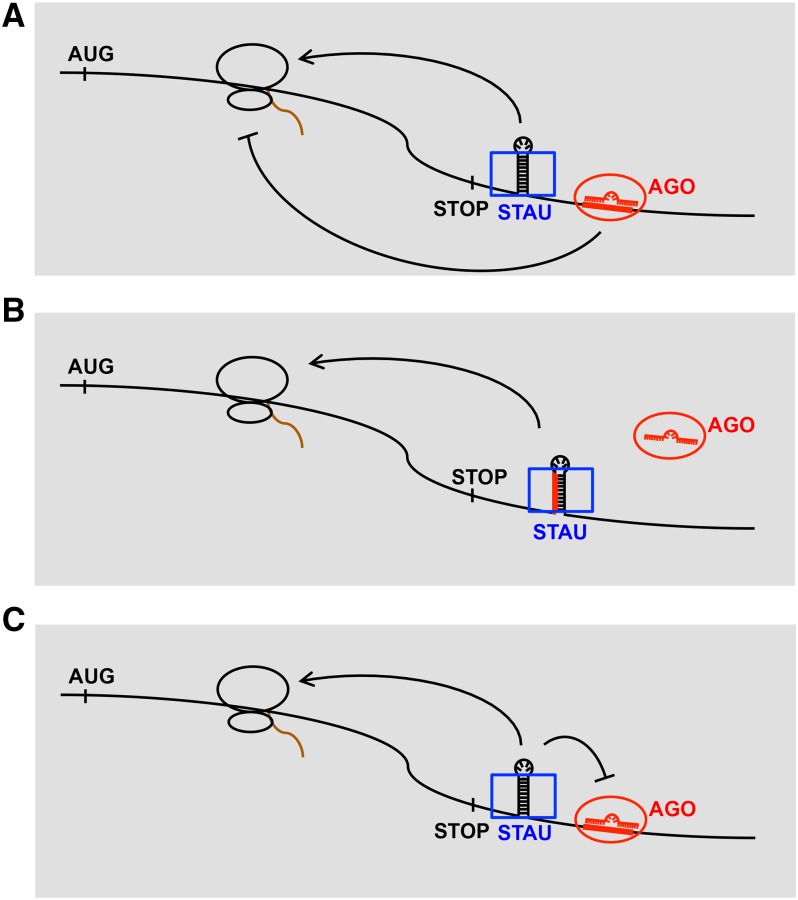
Alternative models for STAU-1-mediated modulation of miRNA activity wherein STAU-1 is proposed to bind to miRNA targets, for example via 3′UTR double-stranded RNA secondary structure. The previously-described translational activation function of Staufen (STAU) could oppose, and hence inhibit the net potency of, miRNA-based translational repression (A); STAU-1 could also affect miRNA activity by binding at (or close to) the miRNA binding site and hence inhibit miRISC binding (B) or activity (C). Blue rectangles are STAU-1 proteins and red ovals are AGO proteins. The red single stranded nucleic acids in AGO proteins represent miRNAs and red lines on mRNAs are miRNA binding sites. Brown curves are newly synthesized protein peptides from the mRNAs.

Various possible molecular mechanisms could be the basis for an opposition of miRNA repression by STAU-1. STAU-1 bound to target mRNA could oppose miRNA activity by exerting an independent translational activation (Figure 7A), or perhaps also by directly inhibiting the binding (Figure 7B) or efficacy (Figure 7C) of miRISC. It should be noted that a target site occlusion model for STAU-1 (Figure 7B) would be similar to the action attributed to other 3′UTR binding proteins (Pumilio, HuR, and Dnd1) that can apparently affect miRNA target accessibility by binding at or near miRNA sites (Fabian and Sonenberg 2012).

All the models proposed here (Figure 7) involve a hypothetical physical interaction of STAU-1 with miRNA targets. Accordingly, one would predict that the miRNA target mRNAs that likely contribute to the phenotypes we observed here (*lin-14*, *hbl-1*, *lin-41*, and *cog-1*) should be recoverable associated with STAU-1 immunoprecipitated from worms of the appropriate developmental stage (embryo for *cog-1*; L1 for *lin-14*, L2 for *hbl-1*, and L4/adult for *lin-41*). We have not tested for these mRNA associations by immunoprecipitation, but a previous study (LeGendre *et al.* 2013) did recover the *let-7* target *lin-41* as enriched in a STAU-1 RNA Immunoprecipitation (RIP) experiment using extracts of *C. elegans* adults, supporting the model that STAU-1 could interact with miRNA targets. LeGendre *et al.* (2013) did not recover *cog-1*, *lin-14*, or *hbl-1* in their RIP experiments, but this is perhaps not unexpected since larval stage extracts were not tested. Consistent with the hypothesis that STAU-1 could interact with the 3′UTRs of these genes by binding to regions of double-stranded RNA (dsRNA) secondary structure to potentiate miRNA activity, these 3′UTRs contain numerous regions predicted to form dsRNA structures, and many of the miRNA complementary sites reside at or near the structured regions (Figure S4). However, the identification of STAU-1 binding sites in *C. elegans* is needed to fully test this hypothesis.

Our small RNA sequencing data indicate that *stau-1* loss-of-function mutation does not affect miRNA biogenesis in general, despite having clear effects on the developmental phenotypes of certain miRNA mutants. Importantly, the levels of the particular miRNAs responsible for those phenotypes were unchanged in *stau-1* mutants. However, we did not examine levels of these miRNAs in specific cell types, therefore, it is possible that STAU-1 could modulate miRNA biogenesis or stability cell type-specifically, and we might not have detected cell type-specific changes of those miRNAs in our RNA samples extracted from whole animals. Indeed, our sequencing data contain a hint that the abundance of some miRNAs could be affected by STAU-1; 11 miRNAs exhibited at least twofold change in levels in the *stau-1* mutant compared to wild type animals (Figure 3C). In such cases, perhaps STAU-1, through its double-stranded RNA-binding activity, can associate with secondary structure elements in miRNA primary transcripts and/or precursors and modulate their processing into mature miRNAs.

Prompted by the finding from a previous study that *C. elegans stau-1* mutants exhibit an enhanced RNAi (Eri) phenotype, and interact genetically with *eri-1* mutation (LeGendre *et al.* 2013), we tested whether an *eri-1* mutation, similarly to *stau-1*, could suppress *let-7* family miRNA mutant’s heterochronic phenotypes. Surprisingly, the *eri-1(mg366)*; *mir-48mir-241(nDf51)* mutant exhibited enhanced heterochronic phenotypes, which is the opposite from the effect of *stau-1*. First of all, this finding indicates that the modulation of miRNA activity by STAU-1 is unlikely to stem simply from an enhanced exogenous RNAi pathway; otherwise, we would have expected that *eri-1(mg366)*, like *stau-1(loss-of-function)*, should suppress *mir-48mir-241(nDf51)* mutant phenotypes. Rather, these findings, particularly the opposite effects of different Eri loci on *let-7* family miRNA phenotypes, suggest important but as yet uncharacterized interactions among RNAi and miRNA pathways in *C. elegans*. ERI-1 is known to be an exonuclease and important for the production of siRNAs in *C. elegans* (Kennedy *et al.* 2004), and a few studies have examined miRNA levels in an *eri-1* loss-of-function context with mixed results, perhaps reflecting differences among experimental systems and/or the particular miRNAs assayed (Lee *et al.* 2006; Duchaine *et al.* 2006; Pavelec *et al.* 2009; Thomas *et al.* 2012). It is clear that further studies are needed to characterize the mechanisms by which ERI-1 affects miRNA activity.

Interestingly, we did not observe any overt miRNA gain-of-function phenotypes for *stau-1* mutations in an otherwise wild-type genetic background, as might be expected for loss of a potent miRNA inhibitor. Rather, the *stau-1* mutants’ miRNA phenotypes were only detected in sensitized genetic backgrounds with compromised miRNA activity. These findings suggest a modulatory effect of STAU-1 on miRNA activity and underscore the importance of miRNA pathways in conferring robustness to biological systems. The modulatory role of STAU-1 on miRNA activity could perhaps be important in refining the posttranscriptional regulation of important miRNA targets and to modulate the efficacy of miRNAs in response to physiological and environmental signals.

In conclusion, our study demonstrates that the RNA-binding protein STAU-1 negatively modulates miRNA activity downstream of miRNA biogenesis, possibly by interacting with the 3′UTR of miRNA targets. These findings reveal an expanded suite of RNA regulatory roles for STAU-1; besides regulating mRNA localization, translation and decay, Staufen can also exert posttranscriptional gene regulation through its engagement with miRNA targets. It should be noted that our results to date indicate that STAU-1 can inhibit the activity of *let-7* family, *lsy-6*, and perhaps *lin-4* miRNAs, but further studies are required to test for similar roles of STAU-1 in opposing the activity of other miRNAs. Moreover, we should not rule out the possibility that STAU-1 could promote the activity of certain other miRNAs, for example through its known role in mediating mRNA decay (Park and Maquat 2013).

## Supplementary Material

Supplemental Material
