# Supplementary material for: Staufen Negatively Modulates MicroRNA Activity in Caenorhabditis elegans
Source: G3 (Bethesda). 2016 Feb 23;6(5):1227–37. doi: 10.1534/g3.116.027300 (PMC4856075; doi:10.1534/g3.116.027300)
Supplement: Supplemental Material [file supp_6_5_1227__index.html]

Staufen Negatively Modulates MicroRNA Activity in Caenorhabditis elegans — Supplemental Material 

# Staufen Negatively Modulates MicroRNA Activity in *Caenorhabditis elegans*

## Supplemental Material for Ren *et al.*, 2016

**Files in this Data Supplement:**

- Table S11 - *C. elegans* strains used in this study. (.pdf, 14 KB)
- File S1 - SI methods. (.pdf, 138 KB)
- Figure S1 - Body morphology phenotype of *stau-1* mutants. (.ai, 13409 KB)
- Figure S2 - Mature miRNA levels in wild type and *stau-1* mutants at the L2 stage. (.ai, 352 KB)
- Figure S3 - ALG-1 and DCR-1 protein levels in *stau-1* mutants. (.ai, 565 KB)
- Figure S4 - Analysis of 3'UTR secondary structures (base-pairing probabilities) surrounding predicted miRNA seed binding sites. (.ai, 473 KB)
- Table S1 - Raw miRNA read counts. (.xlsx, 70 KB)
- Table S2 - Results of differential expression analysis of miRNAs using edgeR. (.xls, 67 KB)
- Table S3 - Raw read count of endo-siRNA, sequences that map anti-sense to coding genes (including 5'UTR, coding exons and 3'UTR regions). (.xlsx, 1578 KB)
- Table S4 - Results of differential expression analysis of endo-siRNA counts using edgeR. (.xlsx, 1467 KB)
- Table S5 - Results of differential expression analysis of endo-siRNA counts on genes in CSR-1 pathway using edgeR. (.xlsx, 368 KB)
- Table S6 - Results of differential expression analysis of endo-siRNA counts on genes in WAGO pathway using edgeR. (.xlsx, 282 KB)
- Table S7 - Results of differential expression analysis of endo-siRNA counts on genes in ALG-3/4 pathway using edgeR. (.xlsx, 185 KB)
- Table S8 - Results of differential expression analysis of endo-siRNA counts on genes in ERGO-1 pathway using edgeR. (.xlsx, 57 KB)
- Table S9 - Raw piRNA read counts. (.xlsx, 1532 KB)
- Table S10 - Results of differential expression analysis of piRNAs using edgeR. (.xlsx, 657 KB)
