# Supplementary material for: Staufen Negatively Modulates MicroRNA Activity in Caenorhabditis elegans
Source: G3 (Bethesda). 2016 Feb 23;6(5):1227–37. doi: 10.1534/g3.116.027300 (PMC4856075; doi:10.1534/g3.116.027300)
Supplement: Supplemental Material [file supp_g3.116.027300_TableS11.pdf]

Table S11. *C elegans* strains used in this study

Name	Genotype
JK4608	*stau-1(q798)* X
JR667	*unc-119(e2498:Tc1)* III; *wIs51* [*scm::gfp* + *unc-119(+)*] V (wild type[§])
N2	wild type
OH3646	*otIs114*; *lsy-6(ot150)* V
VT573	*lin-4(e912)* II; *lin-14(n179)* X
VT1296	*mir-48 mir-241(nDf51) maIs105* V
VT1367	*maIs105* [*col-19::gfp*] V (wild type[§])
VT2361	*otIs114* [*lim-6::gfp* + *rol-6(su1006)*]
VT2485	*wIs51* V; *lin-14(n355n679)* X
VT2692	*let-7(n2853)* X*; maIs105* V
VT2700	*dcr-1(bp132)* III; *wIs51* V
VT2856	*maIs105* [*col-19::gfp*] V; *stau-1(tm2266)* X
VT2857	*mir-48 mir-241(nDf51) maIs105* V; *stau-1(tm2266)* X
VT2858	*otIs114*; *stau-1(tm2266)* X
VT2859	*otIs114*; *lsy-6(ot150)* V; *stau-1(tm2266)* X
VT2862	*lin-4(e912)* II; *lin-14(n179) stau-1(tm2266)* X
VT2870	*stau-1(tm2266)* X (4xbackcross)
VT2876	*dcr-1(bp132)* III; *wIs51* V; *stau-1(tm2266)* X
VT2877	*mir-48 mir-241(nDf51) maIs105* V; *stau-1(q798)* X
VT2878	*wIs51* V; *lin-14(n355n679) stau-1(tm2266)* X
VT2938	*mir-48 mir-241(nDf51) maIs105* V; *eri-1(mg366)* IV
VT3314	*let-7(n2853) stau-1(tm2266)* X*; maIs105* V
VT3354	*stau-1(ma327)* X (2xbackcross)
VT3358	*mir-48 mir-241(nDf51) maIs105* V; *stau-1(ma327)* X
VT3405	*stau-1(ma346)* X (3xbackcross)
VT3406	*mir-48 mir-241(nDf51) maIs105* V; *stau-1(ma346)* X
VT3407	*wIs51* V; *stau-1(ma327)* X
VT3408	*wIs51* V; *stau-1(ma346)* X

[§] These strains were used as wild type in heterochronic phenotype assays.
