# Supplementary material for: Staufen Negatively Modulates MicroRNA Activity in Caenorhabditis elegans
Source: G3 (Bethesda). 2016 Feb 23;6(5):1227–37. doi: 10.1534/g3.116.027300 (PMC4856075; doi:10.1534/g3.116.027300)
Supplement: Supplemental Material [file supp_g3.116.027300_FileS1.pdf]

SI Methods

Microscopy

Young adult animals were mounted on glass slides with 2% agarose pads and anesthetized with 50mM Sodium azide. Images were acquired using a Zeiss Imager Z1 microscope and ZEN software.

Firefly microRNA assay

2µg of total RNA was used per sample for Firefly microRNA assay (http://www.fireflybio.com/), following the manufacturer's standard protocol. Guava easyCyte 8HT was used for analysis. Signals (arbitrary units) were normalized to small nucleolar RNA U6.

Western blot analysis

80µg of protein lysate per sample was used to perform the western blot analysis. ALG-1 protein was recognized by a purified polyclonal antibody (Zou *et al.* 2013) at a 1:1000 dilution. The DCR-1 polyclonal antibody was applied at 1:1000 dilution (Duchaine *et al.* 2006), and anti-α-tubulin antibody (Sigma-Aldrich Cat# T6074) was used at 1:20000 dilution.

3'UTR secondary structure analysis

3'UTR sequences of *lin-41*, *lin-14*, *cog-1* and *hbl-1* were downloaded from (Jan *et al.* 2011). In the cases that multiple 3'UTR isoforms were available, the longest one was taken for the analysis. 3'UTR sequences were then subjected to secondary structure

analysis, using the web server version of RNAfold (http://rna.tbi.univie.ac.at/cgi-bin/RNAfold.cgi). The base-pairing probabilities of the centroid secondary structure were extracted for visualization. Predicted miRNA seed sites on the 3'UTR sequences were downloaded from TargetScan (Jan *et al.* 2011).

SI Reference

Duchaine, T. F., J. A. Wohlschlegel, S. Kennedy, Y. Bei, D. Conte Jr *et al.*, 2006 Functional proteomics reveals the biochemical niche of *C. elegans* DCR-1 in multiple small-RNA-mediated pathways. Cell 124: 343–354.

Jan, C. H., R. C. Friedman, J. G. Ruby, and D. P. Bartel, 2011 Formation, regulation and evolution of *Caenorhabditis elegans* 3'UTRs. Nature 469: 97–101.

Zou, Y., H. Chiu, A. Zinovyeva, V. Ambros, C.-F. Chuang *et al.*, 2013 Developmental decline in neuronal regeneration by the progressive change of two intrinsic timers. Science 340: 372–376.
