# Supplementary figures and images for: Staufen Negatively Modulates MicroRNA Activity in Caenorhabditis elegans
Source: G3 (Bethesda). 2016 Feb 23;6(5):1227–37. doi: 10.1534/g3.116.027300 (PMC4856075; doi:10.1534/g3.116.027300)

**A****Wild type (N2)**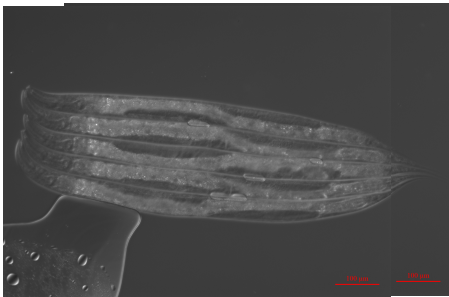**B*****stau-1(tm2266)***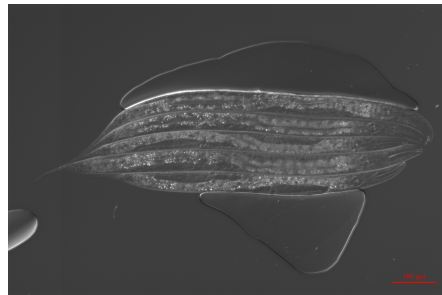**C*****stau-1(q798)***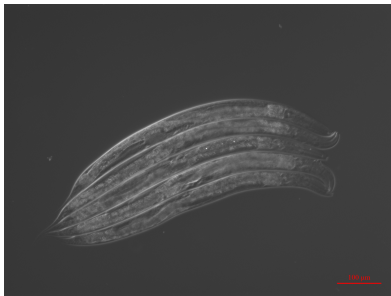**D*****stau-1(ma327)***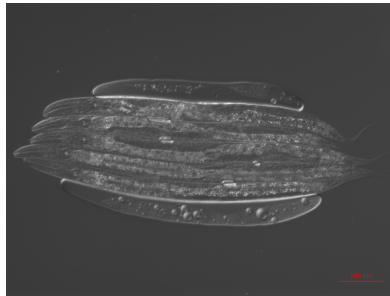**E*****stau-1(ma346)***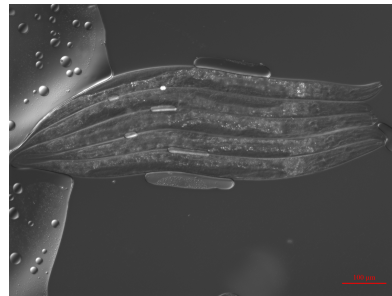

Supplement: Supplemental Material [file supp_g3.116.027300_FigureS1.ps]

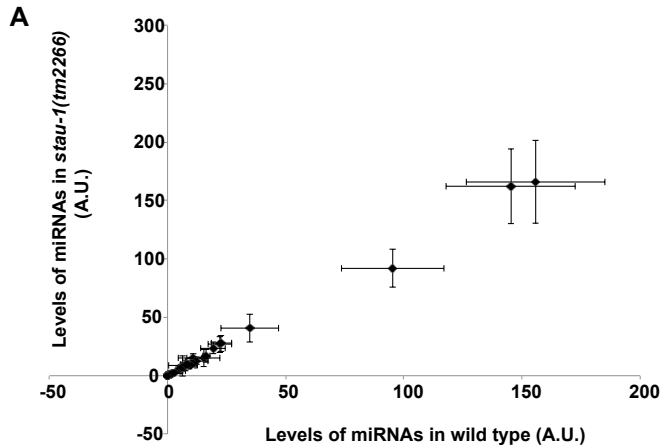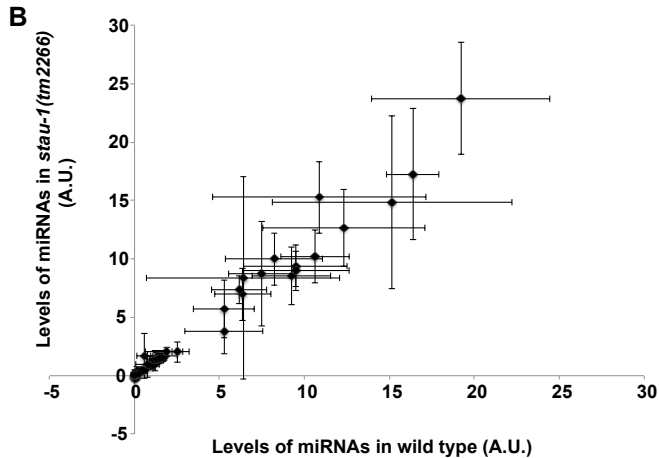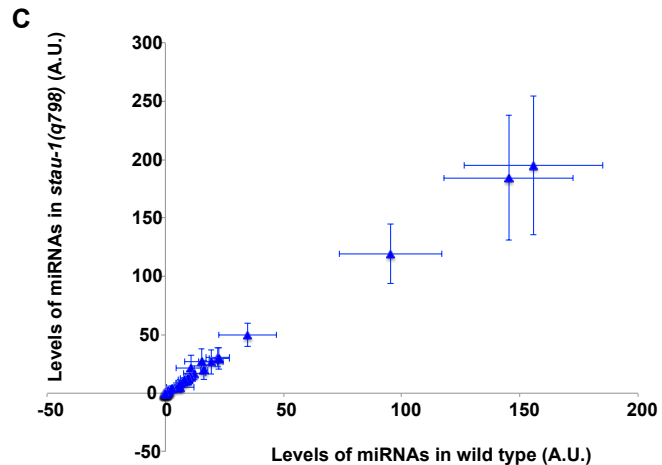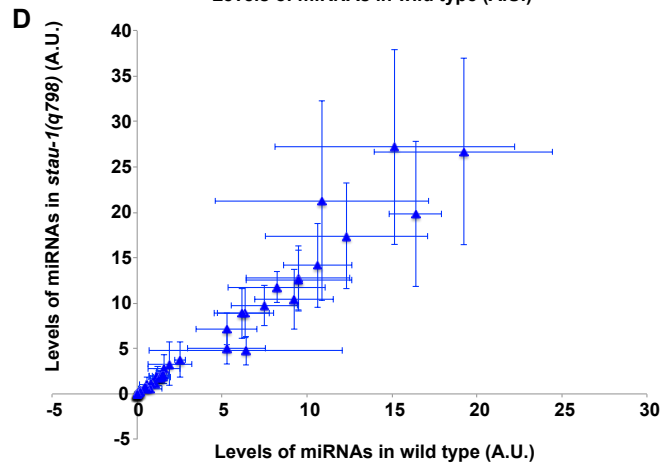

Supplement: Supplemental Material [file supp_g3.116.027300_FigureS2.ps]

**A**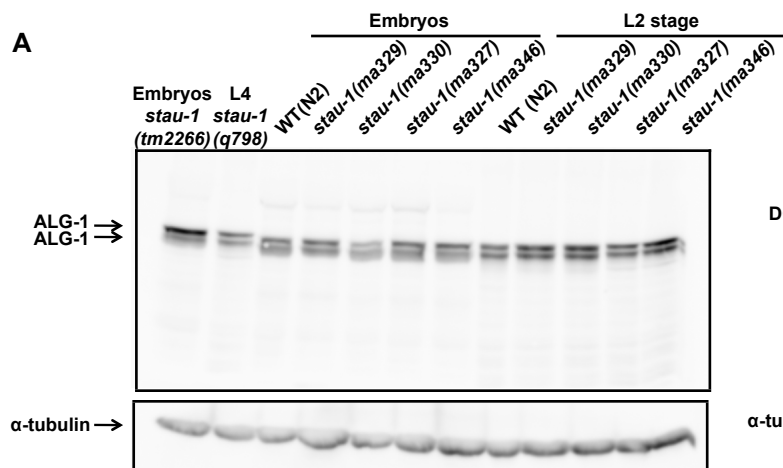**B**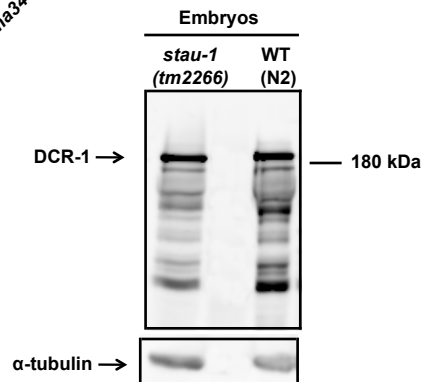**C**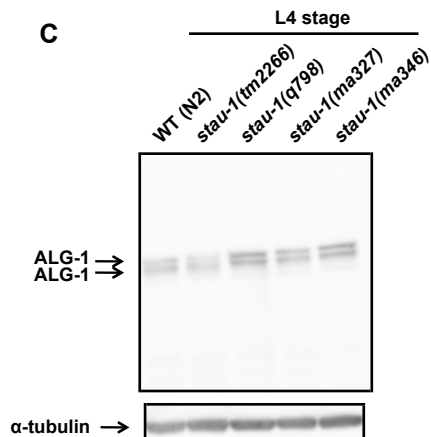**D**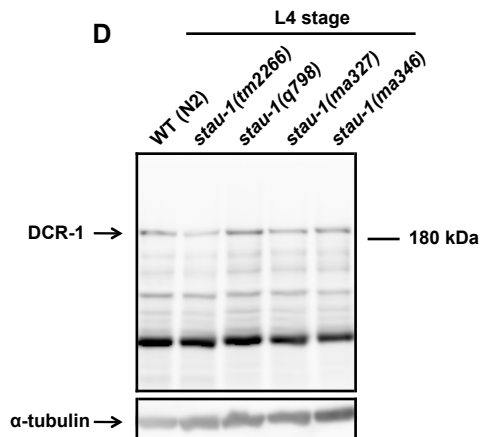

Supplement: Supplemental Material [file supp_g3.116.027300_FigureS3.ps]

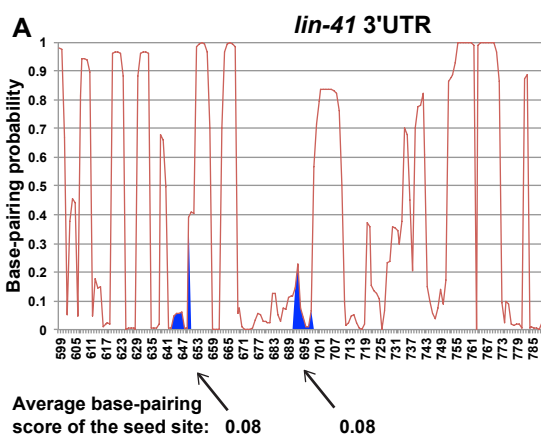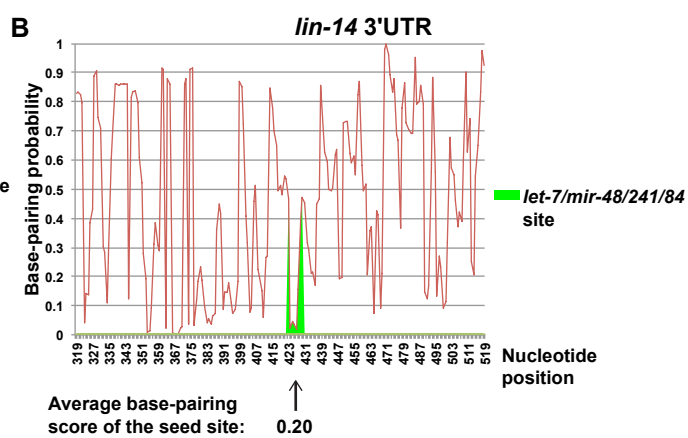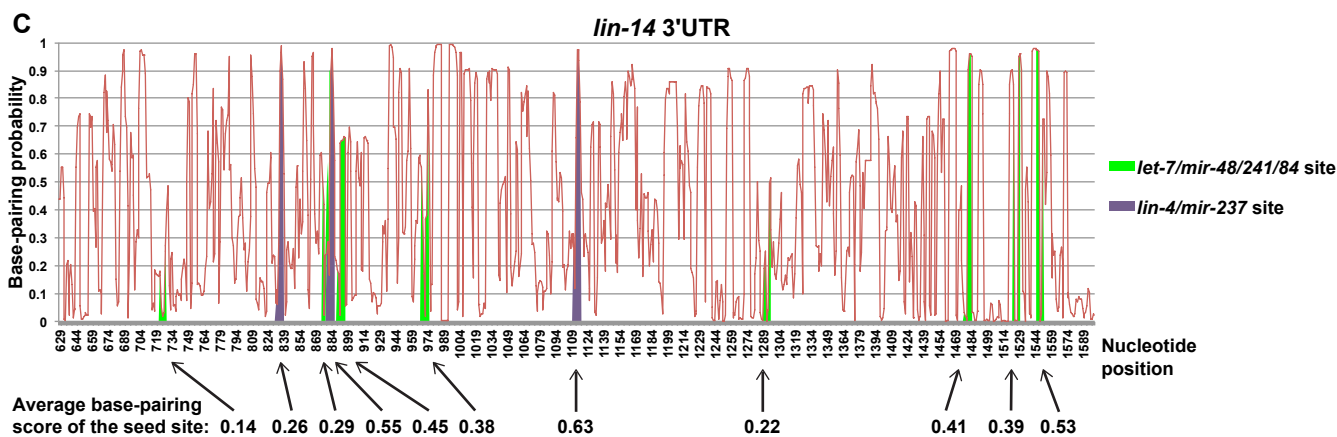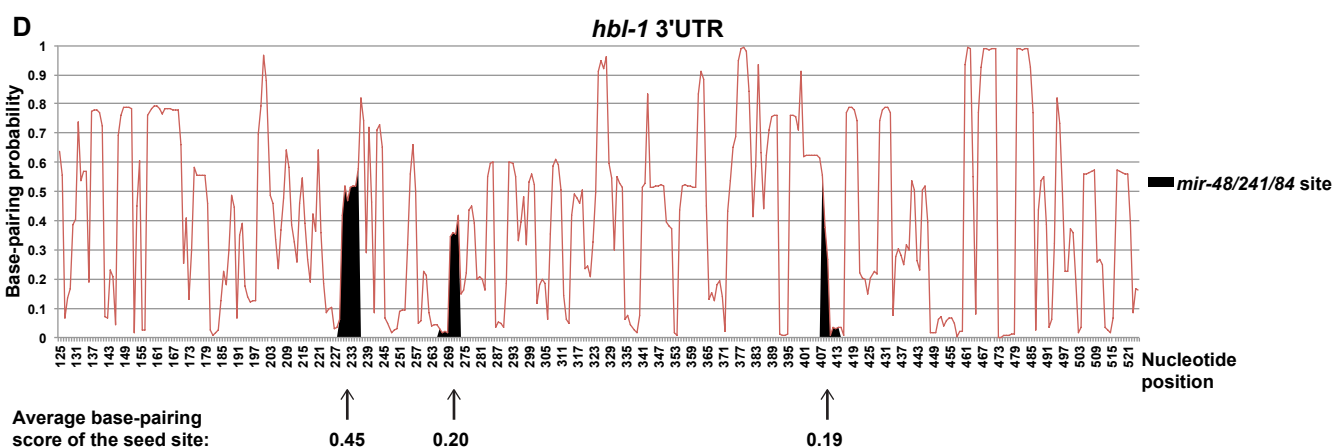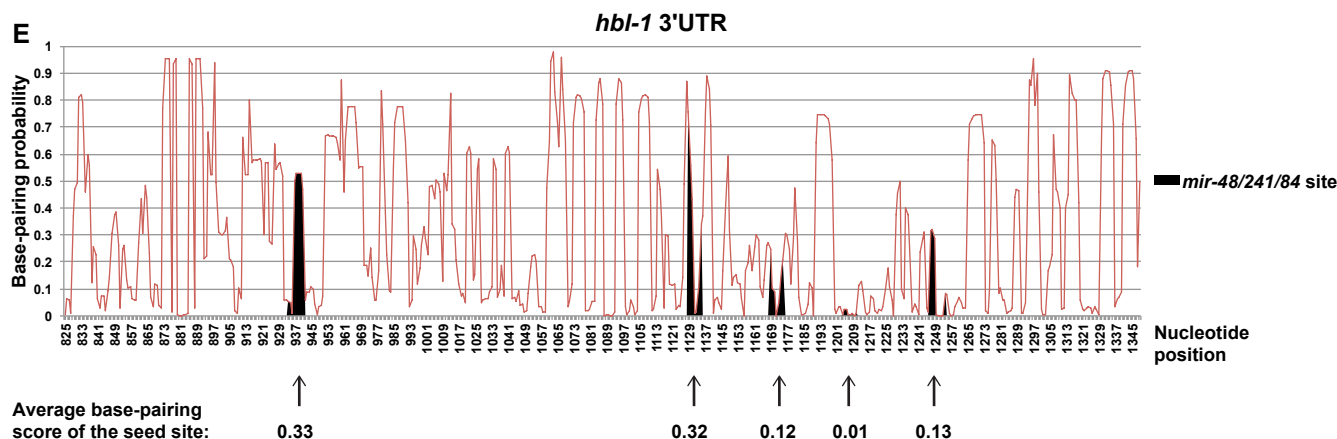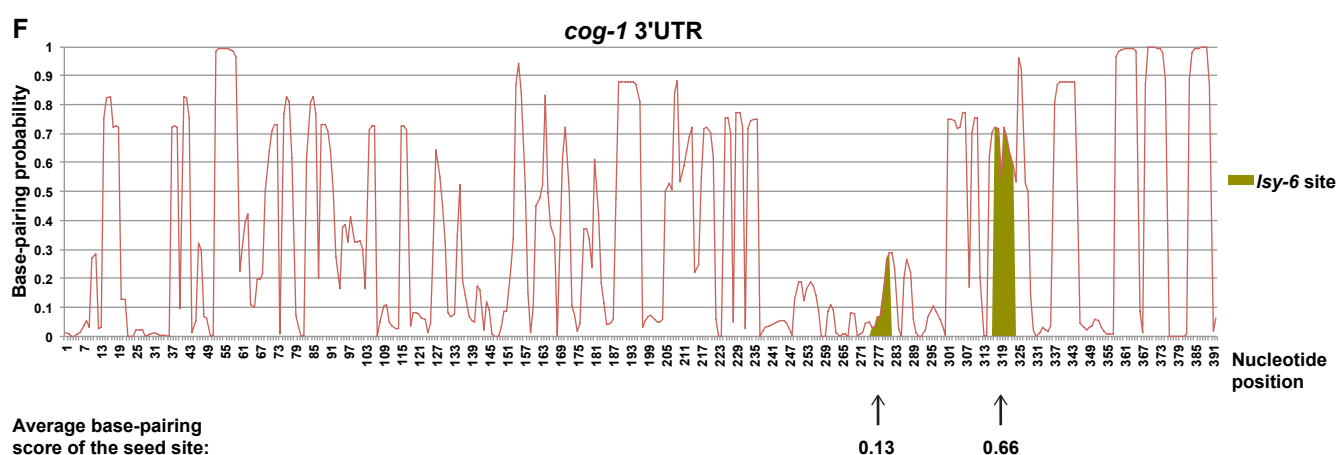

Supplement: Supplemental Material [file supp_g3.116.027300_FigureS4.ps]
